# Sociodemographic and clinical profile of attempted suicide patients

**DOI:** 10.1192/j.eurpsy.2021.1569

**Published:** 2021-08-13

**Authors:** S. Bader, W. Abbes, W. Mahdhaoui, M. Tfifha, I. Rjeb, L. Ghanmi

**Affiliations:** 1 Psychiatry, regional hospital of Gabed, gabes, Tunisia; 2 The Department Of Psychiatry, Hospital of gabes, Gabes, Tunisia; 3 Department Of Psychiatry, regional hospital of gabes, gabes, Tunisia

## Abstract

**Introduction:**

Suicide attempts are common and constitute a serious problem for public health.Thus, it is very important to evaluate risk factors for suicidal behavior.

**Objectives:**

The purpose of this study was to explore the socio-demographic and clinical profile of attempted suicide patients consulting in the psychiatry department in Gabes (southern of Tunisia).

**Methods:**

It was a retrospective descriptive and analytical study covering all patients who had attempted suicide during the period from the 1st of May, 2009 to September 25th, 2020 and who were referred to the psychiatry department in the regional hospital of Gabes. Sociodemographic and clinical data of the patients as well as characteristics of the suicide attemptswere assessed.

**Results:**

Socio-demographic profile of the suicidal consultant in psychiatry department corresponded to a single (73.4%) female (78.8%), with a mean age of 26 years, from an urban area (46%). Suicide attempts were most often by the intentional drug ingestion (67.8%), committed between March and August in 54% of cases. At most of the time, the suicidal person was alone (85%) at home (94%) when he committed his suicidal attempt. He did not communicate his intention to commit suicide in 46% of the cases and only 22 cases (7.9%) notified a person before the suicide attempt and 12.6% afterwards. Suicide behavior was impulsive in 79.5% of the cases and a verbal expression of a desire to die was only noted in 24.5% of cases.

**Conclusions:**

Our results suggest a systematic and specific psychiatric evaluation of any patient who attempts suicide.

**Conflict of interest:**

No significant relationships.

